# CUP-syndrome: Inguinal high grade serous ovarian carcinoma lymph node metastases with unknown primary origin – a case report and literature review

**DOI:** 10.3389/fonc.2022.987169

**Published:** 2022-10-10

**Authors:** Stefano Restaino, Jessica Mauro, Silvia Zermano, Giulia Pellecchia, Laura Mariuzzi, Maria Orsaria, Francesca Titone, Anna Biasioli, Monica Della Martina, Claudia Andreetta, Elena Poletto, Martina Arcieri, Alessandro Buda, Lorenza Driul, Giuseppe Vizzielli

**Affiliations:** ^1^ Department of Obstetrics, Gynecology and Pediatrics, Department of Medical Area DAME, Obstetrics and Gynecology Unit, Udine University Hospital, Udine, Italy; ^2^ Department of Medicine, University of Udine, Udine, Italy; ^3^ Radiation Oncology Department, Academic Hospital of Udine, Udine, Italy; ^4^ Oncology Department, University Hospital of Udine, Udine, Italy; ^5^ Division of Gynecologic Oncology, Michele e Pietro Ferrero Hospital, Verduno, Italy

**Keywords:** high grade ovarian cancer, recurrence, cup syndrome, inguinal lymph node, groin recurrence

## Abstract

**Objective:**

High-grade serous ovarian carcinoma (HGSC) often presents lymph node involvement. According to the paths of lymphatic drainage, the most common site of nodal metastasis is in the aortic area. However, pelvic lymph nodes are also involved and inguinal metastases are less frequent.

**Methods:**

Our report concerns the case of a 78-year-old woman with an inguinal lymph node relapse of HGSC, with the prior positivity of a right inguinal lymph node, after the primary surgery. Ovaries and tubes were negative on histological examination. A comprehensive search of the literature published from January 2000 to October 2021 was conducted on PubMed and Scopus. The papers were selected following the PRISMA guidelines. Nine retrospective studies were evaluated.

**Results:**

Overall, 67 studies were included in the initial search. Applying the screening criteria, 36 articles were considered eligible for full-text reading of which, after applying the exclusion criteria, 9 studies were selected for the final analysis and included in the systematic review. No studies were included for a quantitative analysis. We divided the results according to the relapse location: loco-regional, abdominal, and extra-abdominal recurrence.

**Conclusions:**

Inguinal node metastasis is a rare but not unusual occurrence in HGSC. A reasonable level of suspicion should be maintained in patients with inguinal adenopathy and high CA125 values, especially in women with a history of gynecologic surgery, even in the absence of negative imaging for an ovarian origin.

## Introduction

Ovarian cancer accounts for about 3% of female cancers and represents the fifth leading cause of death in women (1, 2). The incidence of ovarian carcinoma increases with age, peaking during the seventh decade of life (3). More than half of all ovarian cancers are epithelial tumors. Among epithelial ovarian tumors, the serous histotype is certainly the most frequent and includes high-grade serous carcinoma (70%) and low-grade serous carcinoma (<5%). In particular, high-grade ovarian cancer is usually diagnosed in an advanced stage (4, 5)., In two-thirds of all cases, bilateral ovarian involvement is observed, however, in less than 10% of cases, the tumor is restricted to the ovaries. Concerning etiology, one of the most widely accepted hypotheses is that the precursor lesion may originate in the tubal fimbriae (6). There are also cases of hereditary ovarian tumors: approximately 23% of ovarian carcinomas have a hereditary predisposition. Genetically, beyond p53 mutations, high-grade serous carcinomas may present germline and somatic BRCA1 and BRCA2 mutations. The most common hereditary condition is represented by germline mutations in the BRCA1 or BRCA2 genes, that account for 20-25% of high grade serous ovarian cancers (7). An important step in the diagnostic process is molecular profiling, including EpCAM (epithelial cell adhesion molecule), WT1 (Wilms’ tumor protein), MUC16 (cancer antigen 125), MUC1 (cell surface-associated protein), KRT7 (cytokeratin-7), KRT18 (cytokeratin-18) and KRT19 (cytokeratin-19). Apart from its diagnostic relevance, molecular profiling is fundamental in the treatment of advanced ovarian cancer and reveals the need to adopt multidisciplinary strategies to provide the most effective treatments (8). Most patients relapse within 5 years from the first diagnosis, and it would appear that these patients may benefit from cytoreductive surgery in addition to systemic treatment (9). Epithelial ovarian cancer may metastasize intraperitoneally, lymphatically or haematogenously (10, 11). Lymphatic dissemination from the ovary involves the para-aortic and pelvic areas (12), with an estimated incidence of such lesions ranging between 14% and 70% (13). Metastases to pelvic lymph nodes are less frequent than in the para-aortic region (14–16). Probably, in this case, the tumor cells follow the parauterine vessel pathway (uterine artery and vein and iliac artery and vein) within the broad ligament of the uterus (17–19). On the other hand, the possible explanation for the metastatic spread to the para-aortic lymph nodes is that the cells may follow the lymphatic vessels following the ovarian vessels (ovarian artery and vein) within the infundibular-pelvic ligament (20, 21). To the best of our knowledge, there are only a few isolated cases in which metastases to the inguinal lymph nodes from epithelial ovarian tumors have been described (22, 23). The exact mechanism by which tumoral cells spread to the inguinal lymph nodes is still unclear. One possibility is that tumor cells may follow the round ligament of the uterus (24) or the external iliac artery and vein, but it has been argued that the spread to the inguinal lymph nodes should only occur after the blockage of pelvic and para-aortic lymph node stations (25). Considering the peculiarity of this route of spread, the aim of this report is to describe a rare case of groin recurrence of ovarian cancer with negative ovaric and peritoneal biopsies at final pathology.

## Clinical case presentation

In December 2019, a 78-years-old woman with an enlarged right inguinal lymph node was evaluated in another institution. She reported no other symptoms, such as asthenia, abdominal or pelvic pain, or abdominal swelling. She had a medical history, having suffered a pulmonary embolism in 2006, a consequence of deep-vein thrombosis related to her thrombophilic state with a prothrombin mutation. She had no family history of malignancy. Due to a complete utero-vaginal prolapse in December 2017, she underwent a vaginal hysterectomy with preservation of the adnexa. The final histological examination of the surgical sample was negative. Upon reaching menopause, she did not receive any hormone replacement therapy. Her most recent pelvic ultrasound, performed in June 2019, was negative. In December 2019, an enlarged right inguinal lymph node was clinically palpable. An excisional biopsy of the suspicious node in the groin was performed and it showed neoplastic cells related to metastasis of high-grade serous ovarian cancer (Mullerian type), WT1, CK7, CK19, PAX8, ER-positive; TTF1, CDX2, CK20 negative. Considering the histological evidence, the patient underwent a laparoscopic bilateral salpingo-oophorectomy and peritoneal biopsies in January 2020. The final pathology report did not reveal any evidence of disease. The 18FDG PET/CT scan performed in February 2020, showed an increased nodal uptake on the bilateral iliac lymph nodes. From February to June 2020, the patient received six cycles of carboplatin and paclitaxel according to standard protocol. The PET/CT scan images in May 2020 showed a partial response to therapy, confirmed in July 2020. A palpable left inguinal node of about 4 cm was found in November 2020. The PET/CT scan confirmed a suspicion of progression of the disease in the left groin confirmed by a subsequent PET scan in January 2021. At this point, she was brought to our attention and in March 2021 she underwent a diagnostic laparoscopy with peritoneal biopsies. No evidence of disease nor ascites were observed intraoperatively. Thereafter, a left groin lymphadenectomy was performed, which included the removal of an enlarged bulky node of 4 cm. No residual disease was present at the end of surgery. In the final pathology report, the inguinal lymph nodes were positive for high-grade serous ovarian cancer (Mullerian type), whereas all the peritoneal biopsies were negative. No further information about BRCA mutation was available. The woman was considered platinum-resistant since recurrence happened less than 12 months after the end of platinum-based chemotherapy. Therefore, she received treatment of pegylated liposomal doxorubicin (PLD), until September 2021. In October 2021, the PET/CT scan images demonstrated an uptake in the left external iliac lymph node. After multidisciplinary evaluation, the patient was proposed stereotactic radiotherapy for the involved iliac lymph nodes, which was completed in December 2021.

## Materials and methods

We performed a comprehensive search of PubMed and Scopus from January 2000 to October 2021 to identify case reports or case series reporting clinical cases of ovarian cancers with metastatic involvement of inguinal lymph nodes. The research strategy was carried out matching the keywords “ovarian cancer” and “inguinal metastasis” or “ovarian cancer” and “inguinal lymph node metastases” during the period 2000 – 2021. Only English language papers were included. The following information was extracted from the selected articles: the patients’ mean age, the gynecological surgical history, the presenting symptoms, the side of inguinal involvement, the site of the primary tumor and its histology, the surgical treatment, the presence of relapse and their treatment, the use of adjuvant therapies and the CA 125 levels. All studies identified were listed by authors and year of publication. We also traced the references of the articles that had been identified for additional eligible studies. The PRISMA 2020 flow diagram of the selection process is shown in **Table 1**. Two independent investigators (J.M., S.Z.) screened the title and abstracts based on the predefined inclusion criteria. These two authors later independently reviewed the full list of papers, identifying those to be included in the review. Discrepancies were resolved by consensus.

**Table 1:** PRISMA flow chart.

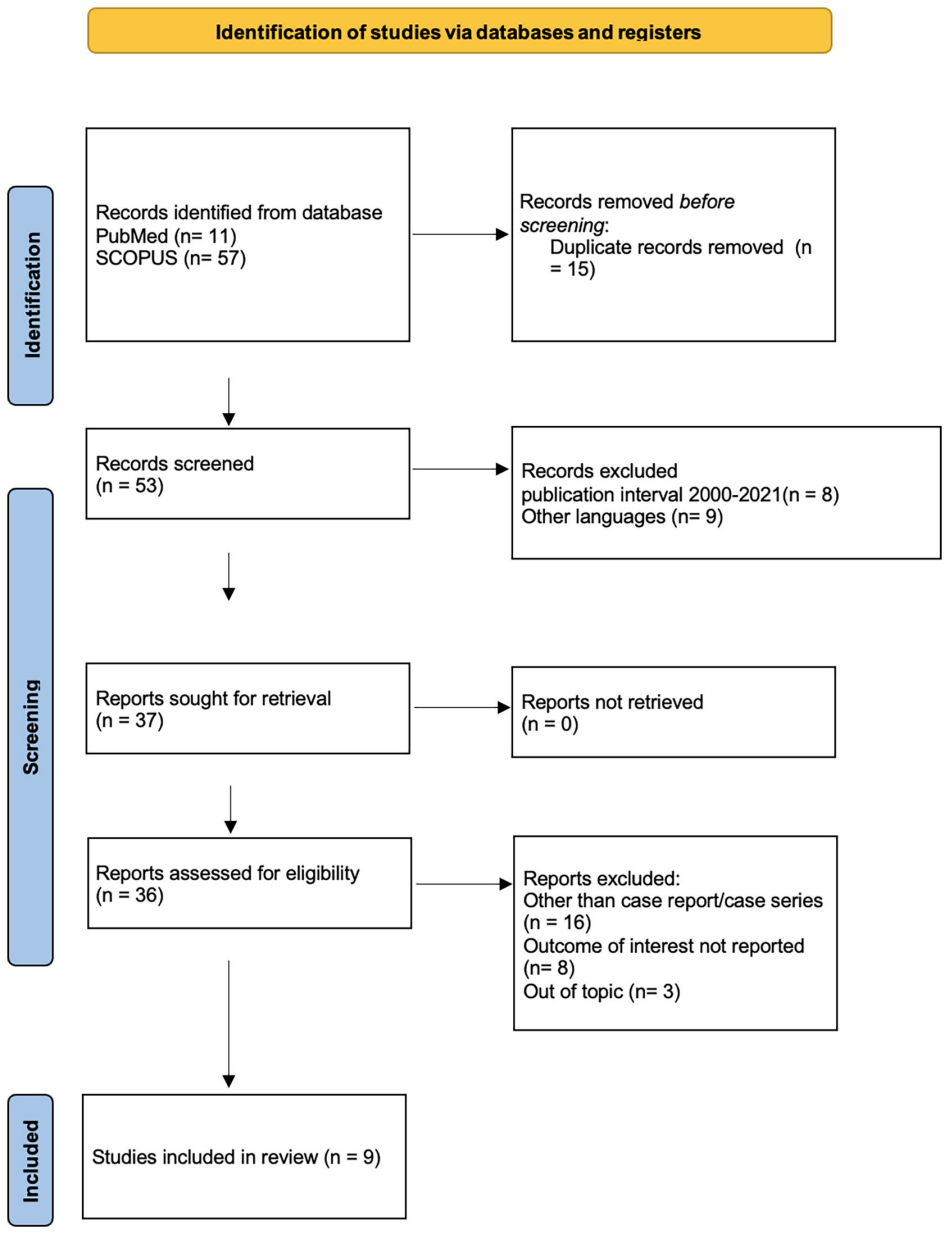

## Results

We identified 68 manuscripts, 10 from Pubmed and 57 from Scopus. After removing duplicates, 53 papers were screened, and 44 papers were excluded because they did not meet inclusion criteria. We analyzed 9 manuscripts. Summary of the characteristics is presented in **Table 2**. Considering the type of studies on this topic, being case reports or case series, we were not able to perform a quantitative meta-analysis. The clinical and pathological characteristics of the subjects included in the studies are summarized in **Table 3**. Twelve women with a median age of 56.1 years old were diagnosed with inguinal lymph node metastasis of ovarian cancer. Initial surgery consisted of lymph node biopsy or fine needle aspiration; bilateral salpingo-oophorectomy was performed in only one patient at the same time. The ovary was the site of the primary tumor in almost all patients (n=10; 83.3%); only in two women the site of the primary tumor remained unknown (n= 2; 16.6%). Inguinal involvement was prevalent on the right side (n= 9; 75%), followed by bilateral forms (n= 2; 16.6%) and more rarely, on the left side (n= 1; 8.3%). Instead, ovarian involvement was generally bilateral (n= 4; 33.3%). Unilateral lymph node involvement was not necessarily on the same side as the ovarian involvement (n= 2). The most common signs and symptoms present were enlarged lymph nodes (n =6; 50%), followed by both abdominal and inguinal pain/and/or swelling in 16.6% of cases; in the minority of cases, patients complained of abdominal swelling (n=1; 8.3%). The gold standard for diagnosis was histological analysis. Most of the cases analyzed by our review showed an increase of the level of CA 125 (n=8; 66.6%). In two cases, this data had not been reported. Only one patient had undergone a hysterectomy in the past, and no other previous gynecological surgery was reported. Adjuvant therapy was performed in eleven patients (91.6%) and only one hadn’t undergone any additional treatment.

**Table 2:** Data of systematic review.

Author	Type of article	Age	First diagnosis	Side	Trtatmeat	Histology	Primary tumor	Ovary side	Relapse	Site	Relapse treatment	Symptoms	Previous gynecological surgery	Adjuvant therapy	CA 125
Ang et al. 2007 (19)	case report	*59*	inguinall LN mtx	right	transcutaneous excision	papillary Serous adenocarcinoma	ovary	left	*/*	*/*	*/*	inguinal pain	*/*	yes	215
Bacalbasa et al. 2818 (26)	case report	46	inguinall LN mtx	bilateral	biopsy	adcriocarcinoma	ovary	bilateral	*/*	*/*	*/*	enlarged right lymph node	*/*	yes	61
Carrabin et al. 2013 (27)	case report	*59*	inguinal! LN mtx	right	transcutaneous excision	borderline tumor	*/*	*/*	*/*	*/*	*/*	enlarged right lymph node	*/*	*/*	*/*
Dam et al. 2021 (28)	case report	62	inguinall LN mix	right	transcutaneous excision	HGSC	not known	*/*	yes	hepatic artery and coeliac trunk lymph ncde	lymph node excision + dlemothmpy	enlarged lymph node	hysterectomy	yes	3628
Ishibashi et al. 2018 (29)	case report	53	inguinall LN mix	left	biopsy	clear cell ovarian carcinoma	ovary	bilateral	*/*	*/*	*/*	enlarged lymph node	*/*	yes	*568*
Manci et al. 2006 (30)	case report	58	inguinal! LN mtx	bilateral	biopsy	adenocaroinoma	ovary	bilateral	*/*	*/*	*/*	enlarged lymph node	*/*	yes	normal
Metwally et al. 2017 (24)	case series	53	ovarian cyst + inguinal LN mtx	left ovary + right lymph nopde	bilateral salpingoopborectomy +LND	HGSC (left) + lymph node mtx	ovary	left	yes	iliac and inguinal lymph nndc	*/*	pelvic massand abdominal pain	*/*	yes	533
		*59*	ovarian cyst + inguinal LN mtx	both ovaries + right lymph mmode	lymph node biopsy	High grade papillary adetiocarcinoma	ovary	bilateral	*/*	*/*	*/*	abdominal pain	*/*	yes	*/*
		65	ovarian mass + lymph node mtx	right ovary +right lymph node	lymph ncde biopsy	HGSC	ovary	right	*/*	*/*	*/*	abdominal swelling	*/*	yes	233
		62	inguinal ma.stasis	right	fine needle aspiration	HGSC	ovary	right	*/*	*/*	*/*	inguinal swelling	*/*	yes	341
Togashi et al. 2020 (31)	case report	43	inguinal metastasis	right	resection	serious adenocarcinomaa	ovary	left	*/*	*/*	*/*	inguinal swelling	*/*	yes	139
Yang et al. 2014 (32)	case report	54	inguinall LN mtx	right	fine needle aspiration	LGSC	ovary	right	*/*	*/*	*/*	inguinal swelln	*/*	yes	normal

**Table 3:** Baseline characteristics, presenting symptoms and type of treatment (n=12).

Mean age	56,1 (SD 6,57)
Gender (male:female)	00:12
Previous ginecological surgery Hvsterectomv	1 (8,3%)
Relaps	2 (16,6%)
Sign and symptoms	
Enlarged lymph node	6 (50%)
Ambdominal pain	2 (16,6%)
lnguinal pain	2 (16,6%)
Inguinal swelling	2 (16,6%)
Abdominal swelling	1 (8,3%)
Origin	
Ovary	10 (83,3%)
not know	2 (16,6%)
Inguinal side	
bilateral	2 (16, 6%)
right	9 (75%)
left	1 (8, 3%)
Ovary side	
bilateral	4 (33, 3%)
right	3 (25%)
left	3 (25%)
not know	2 (16, 6%)
CA 125	
elevated	8 (66,6%)
Normal	2 (16, 6%)
Adiuvant teraphy	
Yes	11 (91, 6%)
No	1 (8, 3%)

## Discussion

Ovarian cancer diagnosis based on the identification of metastatic inguinal lymph node is extremely rare. From the available literature, we found few case reports and manuscripts in which patients with ovarian carcinoma showed positive inguinal lymph nodes at the onset. Therefore, the incidence of inguinal metastasis is not as rare as previously thought. Ovarian cancer shouldn’t be excluded from diagnostic evaluation in patients with isolated groin metastases, especially if associated with higher levels of CA 125.

According to our research, this is the third case described in the literature with inguinal recurrence of serous ovarian cancer with an unknown primary origin. Only one article included in our review describes a clinical case similar to the one we reported (Dam et al., 2021) (28). This finding confirms the rarity of our case report. Our patient first came to our medical attention with an enlarged inguinal lymph node on the right side, and a diagnosis of high-grade serous carcinoma was made after its excision. It is unusual for HGSC to first appear with positive lymph nodes, especially when the ovaries are negative for malignancy on histological examination. Furthermore, in our patient, the first enlarged lymph node was unilateral, on the right side.

Moreover, after 11 months and a favorable initial response to chemotherapy, she developed a contralateral lymph node recurrence, involving the left inguinal region without further metastatic localization. Only two reports, by Metwally et al., 2017 and Dam et al., 2021 describe patients with an episode of relapse, but in both studies, these are single episodes and located in different lymph node sites (24, 28). Additionally, differently from Dam et al., 2021 (28), we performed laparoscopic peritoneal biopsies to exclude primary peritoneal tumors and those were also negative. The report by Kleppe et al., 2015 described three potential pathways of lymphatic drainage in ovarian cancers (25). The round ligament pathway disappears during embryogenesis in most females, but some lymphatic vessels may remain in some women resulting in a pathway for lymphatic spread of ovarian cancer to the inguinal region (25). Moreover, according to Bacalbasa et al.2018, most often, tumor invasion at the inguinal level occurs through the round ligament following a retrograde pathway that is created by tumor occlusion of direct pathways to the pelvic and para-aortic lymph nodes (26). In addition, Metwally et al.2017 suggests the possibility that in one of their cases, a previous intestinal surgery determined anatomical modifications that may have favored the spread of tumor to the inguinal region (24). In this regard, our patient had previously undergone a hysterectomy for a benign pathology, similarly to the patient reported by Dam et al. Therefore, it could be assumed that in these patients, previous surgery could affect the pathways of tumor spread (28). Although the occurrence of ovarian metastases at the level of inguinal lymph nodes remains rare, it may represent an obstacle to diagnosis. A reasonable level of suspicion should be maintained regarding patients having undergone inguinal lymphadenopathies, with elevated CA125 especially if they have a history of previous gynecologic surgery and even if imaging is negative for ovarian disease. It would be useful to perform a diagnostic laparoscopy to define the possibilities of primary extra-ovarian peritoneal malignancy (33). It is also recommended that one or more biopsies be performed to eventually define histology and molecular biology, and also to explore the entire abdominal cavity identifying possible sites of disease (34). In these patients, a multidisciplinary diagnostic and therapeutic pathway should be undertaken, which is essential for a rapid diagnosis and timely treatment. In our specific case, the exact site of origin of the disease remains to be clarified. For this reason, the history of our patient is unique in its progression. On a more general note, looking at the data in the literature, we know that recurrence confined to a lymph node, whatever the location, is a rare event, accounting for 5% of relapsed ovarian cancer (35). An isolated lymph node disease seems to represent a less aggressive pattern of relapse in ovarian cancer and is associated with relatively subdued behavior, without major symptoms.

According to the literature, this attitude may be related to the nature of the tumor cells, which are characterized by a low proliferation rate and an inability to initially develop a peritoneal spread. Also, the microenvironment of the lymph node could keep cells in a dormant state because of the cytokines and T-cells present (36). It seems that this particular pattern of metastatic spread shows a higher load of lymphocyte infiltration upon immuno-histochemical analysis, in particular of CD3 and CD8 cells, rather than extra nodal ovarian cancer relapses. Otherwise, it does not seem to display significant differences in relation to the known genomic subtypes, such as BRCA 1-2 mutations or increase of CCNE1 (37). Further studies on this topic are needed to identify other genetically associated factors related with the development of isolated lymph node metastatic disease.

## Author contributions

SR, GV, LD and AiB contributed to the design and implementation of the research and to the analysis of the results. JM, SZ and GP carried out data collection, processed data and wrote the manuscript. LM, MO, FT, CA and EP were involved in planning and supervised the work. All authors discussed the results and commented on the manuscript. All authors contributed to the article and approved the submitted version.

## Acknowledgments

All authors want to thank Patricia Ann Sawchuk for her valuable contribution in the language revision of the manuscript.

## Conflict of interest

The authors declare that the research was conducted in the absence of any commercial or financial relationships that could be construed as a potential conflict of interest.

## Publisher’s note

All claims expressed in this article are solely those of the authors and do not necessarily represent those of their affiliated organizations, or those of the publisher, the editors and the reviewers. Any product that may be evaluated in this article, or claim that may be made by its manufacturer, is not guaranteed or endorsed by the publisher.
